# Prediction of Steam Burns Severity using Raman Spectroscopy on *ex vivo* Porcine Skin

**DOI:** 10.1038/s41598-018-24647-x

**Published:** 2018-05-02

**Authors:** Lina Zhai, Christian Adlhart, Fabrizio Spano, Riccardo Innocenti Malini, Agnieszka K. Piątek, Jun Li, René M. Rossi

**Affiliations:** 1 Protective Clothing Research Center, College of Fashion and Design, Donghua University, 200051 Shanghai, China; 2 Empa, Swiss Federal Laboratories for Materials Science and Technology, Laboratory for Biomimetic Membranes and Textiles, CH-9014 St. Gallen, Switzerland; 3 Institute of Chemistry and Biotechnology, Zurich University of Applied Sciences, ZHAW, CH-8820 Wädenswil, Switzerland; 4 Key Laboratory of Clothing Design & Technology, Ministry of Education, Shanghai, 200051 China

## Abstract

Skin burns due to accidental exposure to hot steam have often been reported to be more severe than the ones occurring from dry heat. While skin burns due to flames or radiant heat have been thoroughly characterized, the mechanisms leading to steam burns are not well understood and a conundrum still exists: can second degree burns occur without destruction of the epidermis, i.e. even before first degree burns are detected? Skin permeability is dependent both on temperature and on the kinetic energy of incoming water molecules. To investigate the mechanism underlying the injuries related to steam exposure, we used porcine skin as an *ex vivo* model. This model was exposed to either steam or dry heat before measuring the skin hydration via confocal Raman microspectroscopy. The results show that during the first minute of exposure to steam, the water content in both the epidermis and dermis increases. By analyzing different mechanisms of steam diffusion through the multiple skin layers, as well as the moisture-assisted bio-heat transfer, we provide a novel model explaining why steam burns can be more severe, and why steam can penetrate deeper and much faster than an equivalent dry heat.

## Introduction

Injuries due to accidental contact with hot steam can be encountered in employment related situations, such as a rupture of pipes carrying steam^1^ or in high temperature firefighting environment, when sweat that accumulated in the clothing layers evaporates, and heats up due to heat entering the system from the environment^2^. Condensation of hot steam or hot vapor on the cooler skin releases the water’s latent heat of vaporization, rapidly raising the temperature of the skin which will result in steam burns. These types of skin burns are usually very severe and require hospitalization of the patients. However, the mechanisms leading to skin damages through the diffusion of water molecules with a high kinetic energy are not well understood.

Human skin is composed of two primary layers (epidermis and dermis) that protects the inner tissues against external hazards. The outermost thin layer of the epidermis, the stratum corneum (SC), composed of dead cells, is the principal protective component.  It is established, however, that generally skin is not an impermeable barrier.

Based on the fundamental theory of Lane and Blank, there are three pathways for passive diffusion in the epidermis: intercellular, transcellular and through appendages^3,4^. Many *in vivo* studies have shown that moist water permeates into SC of human skin after applications of water patches^5^. Through a combination of *in vivo* confocal Raman microspectroscopy and images of volar–forearm skin captured with the laser scanning confocal microscope, our previous studies^6,7^ showed that the hydration level of the skin and the thickness of the SC increase significantly during uptake of moisture derived from liquid water. Furthermore, when skin temperature is increased, the diffusion of solutes occurs faster^8–10^. For instance^11^, by using a sheet of isolated SC and introducing tritiated water in a temperature-controlled chamber, the water permeability through SC layer can be calculated by analysing liquid scintillation. The results showed that when temperature increases to 70 °C at a relative humidity of 75%, water permeability increases as much as 50 times compared to room temperature. These studies show that permeation and penetration processes will be faster at elevated temperatures.

Skin burn damage mechanisms due to radiant dry heat exposure^12^, combined radiant and convective dry heat exposure^13^, or hot water exposure^14–16^ have been extensively studied, but high temperature water vapor diffusion leading to skin damage has not been analyzed so far. Additionally, through many years of heat and moisture transfer investigations^2,17–21^ on fire fighter clothing under highly moist conditions, it was reported to us that steam burns were frequently more severe than dry ones. Moreover, in one extreme case, the dermis of a firefighter was damaged without any sign of damage in the epidermis layer after steam exposure.

The aim of this study was to explore the variation in water content within skin exposed to steam, to further understand steam burn injuries occurring by moisture-assisted bio-heat transfer mechanism. The changes within *ex-vivo* porcine ear skin in hot steam conditions were investigated by measuring the water content depth profiles using confocal Raman microspectroscopy^22^. Porcine ear skin has been repeatedly reported to have histological and biochemical properties similar to human skin^23,24^, therefore it is considered to be particularly well-suited for permeation studies^24–26^. Using the brick and mortar model of the SC layer (where the bricks are the cells while the mortar represents the lipids), we propose a physical model for the diffusion and penetration of steam. A moisture-assisted bio-heat transfer model is proposed to explain steam burn formation.

## Hydration Profiles upon Water Exposure

**Figure 1:**
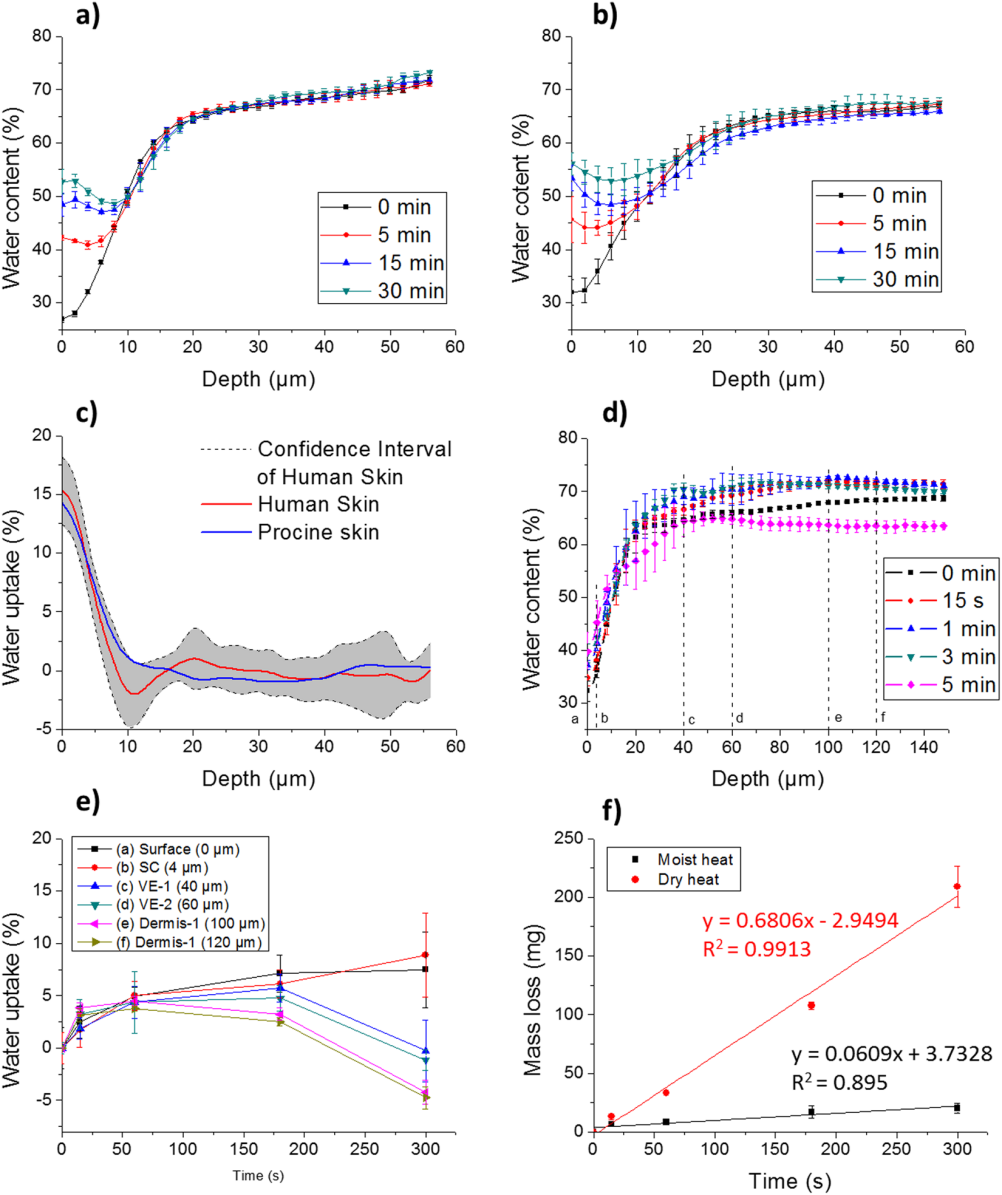
Differences in the skin hydration depth profiles before and after 5, 15 and 30 min water exposure. (**a**) Human skin (*in vivo*) (**b**) Porcine ear skin (*ex vivo*) (**c**) Water uptake profiles of human skin marked with confidence interval and porcine skin after 5 min exposure. (**d**) Depth profiles of water content change after different steam exposure, (**e**) water uptake at different depths as a function of steam exposure times [(**a**) 0 µm (**b**) 4 µm (**c**) 40 µm (**d**) 60 µm (**e**) 100 µm (**f**) 120 µm]. (**f**) Mass loss of skin in steam and dry heat conditions.

In our previous *in vivo* study^6^, a water patch filled with 20 µl of water was applied to the skin of the left arm of a Caucasian woman. The same method was adopted for the porcine ear skin sample (cleaned, trimmed and separated from cartilage) using the same exposure times: 5 min, 15 min, and 30 min. The water depth profiles observed via confocal Raman microspectroscopy from *ex vivo* porcine skin were compared with those obtained from *in viv*o human skin. As shown in Fig. 1a and b, after water exposure, the water content in the SC layer increases for both human and porcine skin. Porcine skin has a slightly higher hydration level from 0 μm to 22 μm and a lower level from 22 μm to 78 μm, and the standard deviation of the measurements is larger. This can be explained by the variability of the porcine ear skin samples. These were taken from several pigs, while the results obtained for human skin^6^ resulted from a single subject. Another explanation could be the slightly altered hydration behaviour of the *ex vivo* model compared to blood perfused *in vivo* skin. Overall, however, the water content depth profiles for the different exposure times obtained for human *in vivo* skin and *ex vivo* porcine skin are in good agreement. The correlation coefficients were higher than 0.98 (*p* < 0.01) for all the exposures tested during the study. Additionally, as shown in Fig. 1c, the typical water uptake depth profile of porcine skin fell into the confidence interval (95%) of human skin, showing the validity of porcine skin as an *ex vivo* model of human skin for water transdermal studies. Further validation of the model was obtained via confocal Raman microspectroscopy and is presented in the supplementary information, see Fig. S1.

## SC and Epidermis Thickness

The changes of the SC thickness of *ex vivo* porcine ear skin after different steam exposure times are presented in Fig. S2. The SC thickness increased from 20.7 ± 2.1 μm to 24.6 ± 1.8 μm after 3 min, at a rate of 1.3 μm/min. This was much faster than the SC increase in thickness measured in human skin when exposed to water at normal ambient temperature (3.7 *μ*m after one hour, from 17.5 ± 2.5 μm to 21.2 ± 3.0 *μ*m)^6^.

According to the results obtained with the fluorescence microscope, the thickness of the porcine ear skin epidermis was 71.4 ± 1.2 μm before exposure to steam. If the same steam diffusion rate is assumed for the SC and the epidermis, the thickness of the latter after 3 min of steam exposure would range from 71.4 μm to 84.9 μm (average 78.2 μm) during exposure.

Therefore, for the interpretation of our results, we assumed that the SC ranged from 0 μm to 22.7 μm (average thickness before and after steam exposure), and the viable epidermis (VE) from 22.7 μm to 78.2 μm. The tissue below a depth of 78.2 μm was assumed to be the dermis. Furthermore, characteristic depths were also selected to better represent different behaviours of the skin layers: 0 µm (surface), 4 µm (SC), 40 µm (VE1), 60 µm (VE2), 100 µm (dermis1) and 120 µm (dermis2), as marked a ~ f in Fig. 1e.

## Steam Exposure

The porcine skin samples were exposed to steam generated by boiling water from a long neck beaker for fixed exposure times: 15 s, 1 min, 3 min and 5 min. The water profiles of the samples after steam exposure were observed via confocal Raman microspectroscopy. Changes in the water content as a function of the depth in porcine ear skin after different steam exposures are shown in Fig. 1d. We observed a strong increase of moisture content in all skin layers during the first 15 s. Then, it stabilized in the dermis until around 150 µm. Depths from 20 µm to 120 µm showed the largest differences in the water content. Between 15 s and 3 min, the changes of water content were generally very small. At 5 min of exposure, however, the water content of the skin tissue decreased dramatically.

For a detailed study of the water changes as a function of time and depth, the water uptake in the previously defined layers as a function of steam exposure time are illustrated in Fig. 1e. In order to show the statistical significance of water changes, the water content from 0 s to 15 s, 15 s to 1 min, 1 min to 3 min, and 3 min to 5 min, was compared using the unpaired student’s t-test (Origin® 2015, OriginLab Corporation). 5 depth profiles were recorded for each skin samples at different locations and for each skin sample, 3 replications were made. Therefore, 30 data points were used to perform one unpaired student’s t-test. The t-test results are shown in Table 1. Significant changes (*p* < 0.05) are marked with “*”, an increase of water is marked with “↑” and a decrease as “↓”.

**Table 1:** Probability results (P-value) of unpaired student’s t-tests on water uptake between different exposure times at different layers.

Water uptake	(a) Surface	(b) SC	(c) VE1	(d) VE2	(e) Dermis1	(f) Dermis2
0 s vs. 15 s	0.0686↑	0.0655↑	0.0007*↑	0.0004*↑	4.0 × 10^−7^*↑	3.3 × 10^−5^*↑
15 s vs.1 min	0.0649↑	0.0028*↑	0.0029*↑	0.2721↑	0.3691↑	0.3894↑
1 min vs. 3 min	0.1335↓	0.2430↑	0.1592↑	0.7020↑	0.0722↓	0.0203*↓
3 min vs. 5 min	0.8259↓	0.0759↑	0.0005*↓	9.1 × 10^−8^*↓	2.9 × 10^−10^*↓	8.7 × 10^−10^*↓

*Significant changes (*p* < 0.05).

(**a**) 0 µm (**b**) 4 µm (**c**) 40 µm (**d**) 60 µm (**e**) 100 µm (**f**) 120 µm.

Figure 1e and Table 1 show that 15 s exposure to steam was sufficient to increase the water content in SC and another significant increase was observed between 15 s and 1 min. VE and dermis significantly absorbed water for the first 15 s, while the changes were not significant after 1 min for VE, and after 15 s for dermis. Dermis started to lose water after 1 min, which was earlier than epidermis (from 3 min on). At 5 min of exposure, a significant decrease of water content occurred in both VE and dermis and a clear shrinkage of the diameter of the skin sample was observed. The thickness in the middle of the skin sample increased, denoting its denaturation. However, in the SC, we observed a continuous moisture uptake during all the 5 min of exposure.

## Mass Loss

The porcine skin samples were weighted on a scale before and after each exposure to steam or dry heat. As shown in Fig. 1f, in both dry and moist heat conditions, the skin samples lost mass. The skin samples lost mass linearly in time with a rate of 0.68 mg/s upon exposure to dry heat, much faster than in moist heat (steam) condition (0.06 mg/s).

Two competing mechanisms contributed to the mass change: steam uptake and water evaporation from sample tissue, especially on the back side of the sample. After dry heat exposure, a visual inspection showed that the skin samples had become dry and stiff. On the contrary, when exposed to steam, the skin samples remained moist and elastic. Therefore, the mass loss difference between these two conditions is mainly due to the evaporation of water from skin tissue.

The interpretation of the results is based on the model described in the methods part. The model illustrates the mechanism of steam diffusion and penetration through the skin, introducing a moisture-heat transfer process, which influences the formation of the burn injury.

## Steam Penetration and Diffusion

**Figure 2:**
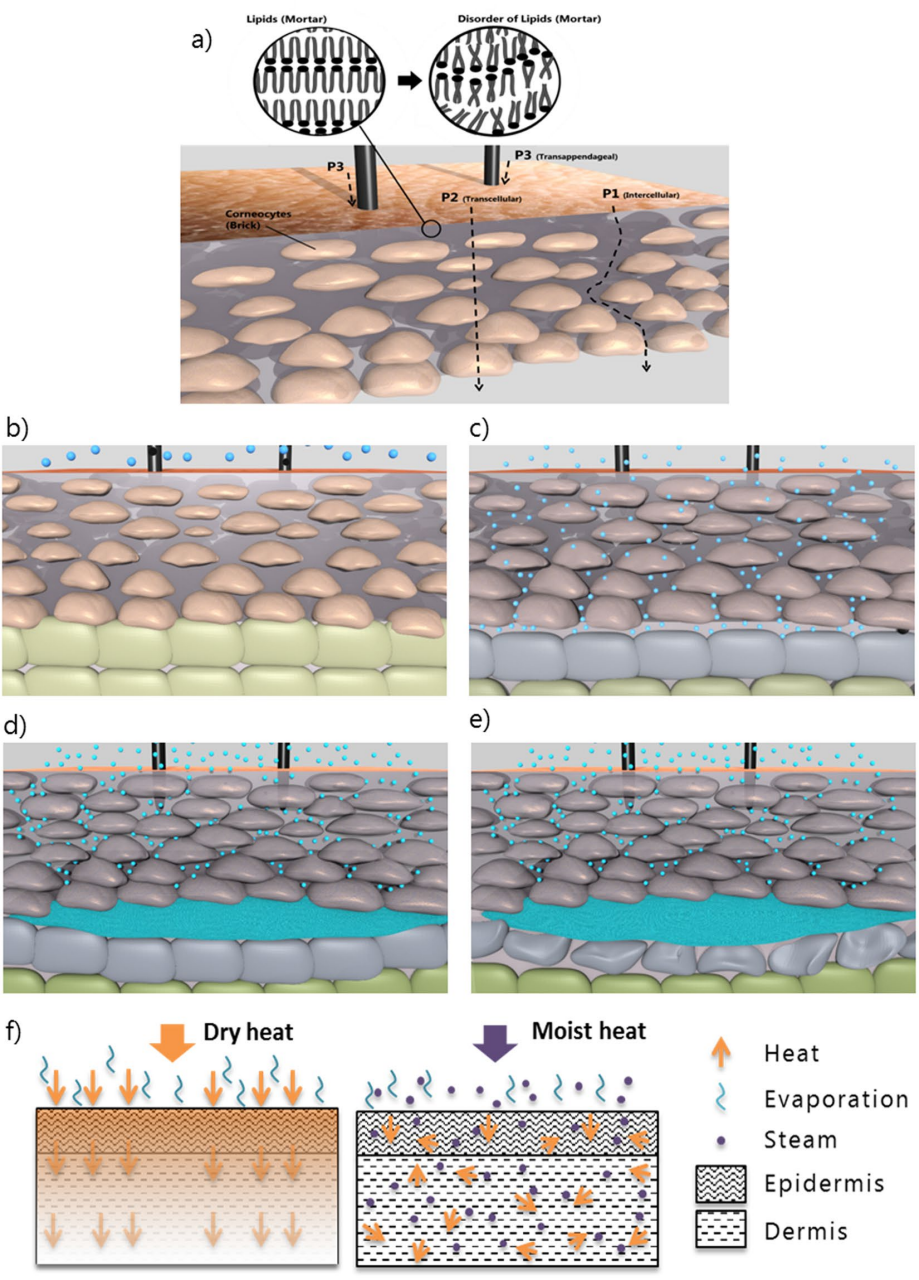
Model of steam diffusion, penetration through the skin and illustration of the second degree burn model without first degree burn. (**a**) Schematic of the different penetration pathways of steam. Detailed illustration of the penetration of steam through the skin: (**b**) Steam in contact with the stratum corneum. (**c**) Penetration of the steam through the 3 pathways and accumulation of steam at the skin barrier (dermis). At this point, corneocytes are starting to swell slowing down the penetration of steam. (**d**) Accumulation of steam and condensation of steam. (**e**) Accumulation of hot water due to condensation of steam and degradation of the first layers of Dermis. (**f**) Schematic of different mechanisms for heat transfer in dry heat condition and in moist heat condition.

As shown in Fig. 1d and e and Table 1, there is a significant increase in water in VE and dermis during the first 15 s of steam exposure. This can be explained by intercellular diffusion: steam penetrates between the cells of the SC (pathway P1, Fig. 2a) and condensates in deeper tissues. It is well accepted that the SC layer^27–29^ is the main barrier of the skin. However, according to Gregor, the SC layer is a porous medium which has a large number of quasi-semi-circular pathways^3,30^ (defined as P1 in our model – Fig. 2). P1 is made of free-volume voids created by random fluctuations in the alkyl chain packing of the lipids (See Fig. 2a Lipids). Researchers have shown that there is a strong correlation between water permeability and SC lipid alkyl-chains disorder (i.e. free volume, respectively pore size)^11,28^. The pore sizes of SC (i.e. the pathway-width) vary from less than 0.4 nm up to approximately 100 nm^30^ (typically of the order of 10 nm). Therefore, most of the pore sizes are much bigger than the size of a water molecule (≈0.3 nm in diameter)^31^. When temperature increases, according to previous studies^9,11^, lipids become less viscous and partially melt leading to a higher degree of disorder of the alkyl chains (See Fig. 2a Disorder of Lipids). It can be hypothesized that this disorder of the alkyl chains results in even larger free-volume voids, which will increase steam penetration. As shown by Ogiso *et al*.^9^, the epidermal tissue underlying SC does not contribute to the barrier properties of the epidermis. As the steam diffuses through the epidermis, nonetheless the water molecules lose energy through heat transfer to the colder tissue, leading to their condensation in both the epidermis and dermis (Fig. 2).

The water uptake in the SC is explained by the transcellular pathway (P2), as steam diffuses through the corneocytes (Fig. 2a and c). As shown in Table 1, the water content increased significantly in the SC layer during the 5 min exposure, leading to an increase of the SC thickness of 3.9 μm after 3 min of exposure to steam. This is in good agreement with previous studies showing that the thickness of the SC layer could approximately increase by 25% in thickness^32,33^. According to Bouwstra *et al*.^34^, the increase of the SC layer is due to the swelling of corneocytes in the direction perpendicular to the skin surface after water exposure (Fig. 2d). However, we observed a much faster diffusion of water (steam) compared to normal ambient temperature (3.6 μm after 60 min)^6^. This supports previous studies stating that the diffusion of solutes occurs faster at high temperatures^8–10^.

The swelling corneocytes in turn affect the free-volume voids, i.e. the transcellular pathway (P2) affects the intercellular one (P1). To be specific, these two competing mechanisms influence the void spaces: the exposure to heat increases the void spaces while the swelling of the cells through steam absorption reduces them (Fig. 2). This is supported by our results shown in Table 1.

The water uptake in SC was not significant during the first 15 s (unlike in VE and dermis), suggesting that heat contributed to increase the intercellular void sizes and that steam transfer through pathway P1 was more important than P2 during the first seconds of steam exposure. After 15 s, however, the water content in SC and upper VE increased significantly while there was no significant water uptake in the deeper layers (60 µm up to 120 µm) indicating swelling of corneocytes leading to a reduction of the void spaces and thus of the water free path. The water uptake and thus the swelling of the cells stopped to be significant between 1 and 3 min of steam exposure indicating that the corneocytes gradually became saturated.

## Moisture-Assisted Bio-Heat Transfer and Steam Burn Formation

After 5 min of steam exposure, as shown in Fig. 1d, there was a significant drop of water content from 20 µm to 120 µm of skin. Additionally, Fig. 1f indicates that water loss started from the beginning of the exposure to steam. This further supports our moisture-assisted bio-heat transfer model. As shown in Eq. 5, steam penetration and diffusion generate a positive volumetric heat flow (*q*_*st*_) while water loss leads to a negative volumetric heat flow (*q*_*wl*_). It was not possible to perform the differential numerical calculation due to insufficient detailed data, such as the in depth profiles of steam penetration and water loss. However, the accumulated heat, *Q*_*st*_, *Q*_*wl*_ and *ΔQ* in a period of time can be calculated using Eq. 1–3.

1 $${Q}_{st}={{\rm{\Delta }}}_{{\rm{v}}{\rm{a}}{\rm{p}}}H{m}_{st}+{C}_{p}{m}_{st}{\rm{\Delta }}T$$

2 $${Q}_{wl}={{\rm{\Delta }}}_{{\rm{v}}{\rm{a}}{\rm{p}}}H{m}_{wl}+{C}_{p}{m}_{wl}\,{\rm{\Delta }}T$$

3 $${\rm{\Delta }}Q=({Q}_{st}\,{\textstyle \text{-}}\,{Q}_{wl})=({{\rm{\Delta }}}_{{\rm{v}}{\rm{a}}{\rm{p}}}H+{C}_{p}{\rm{\Delta }}T)({m}_{st}-{m}_{wl})=({{\rm{\Delta }}}_{{\rm{v}}{\rm{a}}{\rm{p}}}H+{C}_{p}{\rm{\Delta }}T){\rm{\Delta }}m$$

where Δ*Q* is the heat accumulation in tissue induced by moisture, (J). Δ*H* is the enthalpy of phase changes from water to vapour, and is a constant value, (J/kg). *C*_*p*_ is the heat capacity of water, (J/kg · K). *m*_*st*_ is the water mass gained due to the steam penetration process, (kg); *m*_*wl*_ is the water mass lost arising from diffusion outside the skin layer from the bottom, and top layer, (kg); Δ*m* is the water mass variation, calculated by (*m*_*st*_ − *m*_*wl*_), indicating the change in the total amount of water in the system, (kg). Δ*T* is the temperature variation of water due to heat transfer to skin, (K).

To simplify this analysis, we assume that temperature variation (Δ*T*) of condensed water and evaporated water is the same, based on Eq. 3, and that the weight change of a layer is only due to condensation/evaporation effects, with no liquid water transfer between the layers. Then the water mass variation Δ*m* (*m*_*st*_ − *m*_*wl*_) determines the moisture-induced heat variation Δ*Q*. By extracting data from the measurements presented in Fig. 1d, the water mass variations can be calculated for epidermis (average variation at 40 µm and 60 µm) and dermis (average variation at 100 µm and 120 µm). Water mass variations at four exposure stages are shown in Table 2.

**Table 2:** Water mass variation (%) at epidermis and dermis during the four stages.

Δ*m*	I (0 s–15 s)	II (15 s–1 min)	III (1 min–3 min)	IV (3 min–5 min)
Epidermis	2.5	1.8	0.9	−6.0
Dermis	3.5	0.7	−1.3	−7.3

As shown in Table 2, the mass increased more in the dermis than in the epidermis in stage I, while it started to decrease in stage III. This indicates that more heat was generated in the dermis in stage I but more heat was removed in stage III than in the epidermis. This uneven heat variation for the two layers caused uneven temperature gradient. As burn injuries are dependent on both the exposure duration and the temperature, therefore, the risk, that the dermis got damaged by the heat was higher than the epidermis in stage I, which would imply that the dermis could suffer from a skin burn earlier than the epidermis. First degree burns are characterized by the damage of the epidermis, while second and third degree burns describe a damage of the dermis. Due to steam penetration and diffusion as well as water evaporation, the order of first/second/third degree burns could be changed when the temperature of the dermis increases faster or stays for longer times above 44 °C, which explains why steam burns are often more severe than would be the case in equivalent heat load dry heat burn injuries.

For dry heat conditions, the model can be simplified by removing the moisture-assisted volumetric heat flow terms (*q*_*st*_ and *q*_*wl*_) from Eq. 5. In this model, heat diffuses from layer to layer, showing a temperature gradient from the top layer to the deeper tissue. As a result, first degree, second degree, and third degree burns will be induced in the normal order. In other words, the moisture-assisted heat transfer parts explain the disorder of burn injury degree in steam condition.

In Fig. 1f, the mass loss was due to evaporation of water on both sides of the pig skin samples. In reality, the mass loss from the dermis would obviously not occur, but we can pertinently assume that the evaporation from the SC was much higher in dry heat condition than in steam. This is because during exposure to steam, the atmosphere at the skin/air interface is saturated with water vapour and the high water vapour partial pressure prevents the evaporation of the moisture contained in the skin tissue. Due to less water evaporation and relative evaporative cooling, skin tissue stores more heat in steam conditions which makes steam burns even more serious.

However, as described by Blank, SC resists heat damage, tolerating temperatures as high as 60 °C for several hours^9^. Instead of becoming dry and stiff, in steam condition, the hydration of the SC continues to increase even at the end of steam exposure (Fig. 1d).

The aim of this study was to explore the water content variation in *ex vivo* porcine skin before and after steam exposure to further understand steam burn formation by moisture-assisted bio-heat transfer mechanism.

Our study showed that in steam conditions with a heat intensity of 4.3 kW/m^2^, the water content of porcine ear skin significantly increased in both VE and dermis during the first 15 s. These weight changes became non-significant for VE after 1 min exposure, and already after 15 s for the dermis. In the SC, we observed a continuous moisture uptake until the end of 5 min exposure. This can be explained by a model of steam diffusion and penetration through the skin. Steam penetrates the skin through the intercellular pathway and diffuses through the transcellular pathway. At the same time, SC thickness increases with a rate of 1.3 μm/min (swelling of corneocytes), which in turn slows down the penetration of steam. We proposed a moisture-assisted bio-heat transfer model of skin in steam condition, which accounted for the heating effect due to steam diffusion and penetration as well as the cooling effect due to evaporation of water. As water evaporation from the skin is lower in steam condition (water loss rate of 0.06 mg/s) than dry heat condition (0.68 mg/s), the model showed higher skin heat storage in steam condition. It suggests that the moisture-assisted heat transfer accelerates burn formation and can also lead to a change in the order of the burn injury in the epidermis and dermis. This hypothesis is consistent with the disorder of first and second degree burn injury in steam conditions reported by firefighters from practical cases.

Our study introduces an innovative approach explaining how steam burns are induced, and explains why steam burns can be so much more severe than would be the case in equivalent heat load dry heat burn injuries. Our model is useful for addressing the dangerous steam conditions in daily life and is especially important for workers who are often confronted with steam hazards.

There is a complex process of dynamic water exchange occurring in steam conditions. The water, temperature, and heat variation induced by moisture are still not quantitatively calculated for each layer. Such quantification would require further investigations, using for instance heavy water as a steam source to discriminate the internal and external source of water. By obtaining the in depth profiles of steam penetration and water loss, and a detailed track of the initial/boundary condition of the porcine sample, it would be possible to numerically model the temperature field and the steam movement within the skin.

## Methods

### Model of steam diffusion and penetration through the skin

A physical model is proposed in order to explain steam diffusion and penetration through the skin. SC is composed of alternating layers of flat, protein-rich cells surrounded by an extracellular lipid matrix. This organisation is modelled as “brick and mortar”. As shown in Fig. 2, the corneocytes are described as the bricks and the lipid bi-layers are the mortar. Furthermore, according to Lane and Blank, there are three path ways for passive diffusion in epidermis (Fig. 2): (1) P1: between the cells of the SC (intercellular pathway), (2) P2: through the cells of the SC (transcellular pathway), and (3) P3: through appendages such as hair follicles and sweat glands^3^ (transappendageal pathway). We hypothesize that hair follicles which are considered to play only a minor role in the transport of water^35^ are a negligible pathway for hot steam.

Therefore, pathway P1 and P2 are the main concerns in our model and we hypothesize that there are three mechanisms occurring when the skin is exposed to steam:

1. Steam penetrates into skin through P1. In intense heat condition, the free-volume voids of the lipid lamellae (Mortar) increase to allow the penetration of steam to go through this porous medium. And the moisture vapour finally condensates in the deeper tissue.
2. Steam diffuses through P2. Similarly as during water diffusion, steam vapor can be absorbed by corneocytes (brick), and we assume that during this process, the corneocytes swell.
3. As an interdependent mechanism, the swelling corneocytes in turn decrease the free-volume voids.

Therefore, there would be two conflicting mechanisms: the exposure to heat increases the void spaces while the swelling of the cells through steam absorption reduces them.

### Model of steam burn formation

According to burn injury theory^14^, burn damage is assumed to be the result of heat-induced changes in proteins' structure. The bio-heat transfer inside of skin is often described by using Pennes bioheat equation^36,37^:

4 $$\rho {C}_{p}\frac{\partial T}{\partial t}=k\frac{{\partial }^{2}T}{\partial {x}^{2}}+{\omega }_{b}{\rho }_{b}{C}_{p,b}({T}_{b}-T)+{q}_{met}$$

where *ρ* (kg/m^3^), *Cp* (J/kg · K), *k* (W/m · K), and *T* (K) are the density, specific heat capacity, thermal conductivity, and temperature of skin tissue, respectively; *ρ*_*b*_, *C*_*p,b*_, *T*_*b*_ are the density, specific heat, and temperature of the blood, respectively*; ω*_*b*_ (m_b_^3^/_s_·m_s_^3^) is the blood perfusion rate per unit volume. *q*_met_ (W/m^3^) is the metabolic volumetric heat flow.

In steam condition, we propose an adaption of the Pennes bioheat equation for the moisture-assisted heat transfer^38^. In the new equation (Eq. 5), the cooling effect due to evaporation of water from the skin tissue and the heating effect due to steam diffusion/condensation and penetration are added. Therefore, the different mechanisms for heat transfer in dry heat and moist heat conditions are schemed in Fig. 2f.

5 $$\rho {C}_{p}\frac{\partial T}{\partial t}=k\frac{{\partial }^{2}T}{\partial {x}^{2}}+{\omega }_{b}{\rho }_{b}{C}_{p,b}({T}_{b}-T)+{q}_{met}+{q}_{st}-{q}_{wl}$$

where *q*_st_ (W/m^3^) is the volumetric heat flow generated by steam penetration and diffusion, *q*_wl_ (W/m^3^) is the volumetric heat flow removed by water loss.

Excessive heat leads to structural changes of proteins^39,40^. Proposed by Moritz and Henriques^14^, protein denaturation can be described by the first-order Arrhenius rate equation.

6 $$\Omega ={\int }_{0}^{t}P\,\exp (\,-\,{\rm{\Delta }}E/RT)dt$$

where *Ω* is the burn injury parameter; *P*(s^−1^) is a pre-exponential term, and *R* (8314.5 J/mol · K) is the universal gas constant; *ΔE* (J/mol) and *T* (K) are the activation energy and absolute temperature of skin; *t* is the total time for which *T* is above 317 K.

Based on Eq. 6, burn injuries are only dependent on the duration and temperature profiles when the temperature is above 44 °C, but are independent of the depth. As the burn degree is determined by the depth of denatured protein, we made the following assumption: If the temperature of dermis increases faster or stays longer above 44 °C than the one of the epidermis, the order of first/second/third degree burn can be changed.

### Sample preparation

The fresh pig ears were obtained from a local slaughterhouse (Zett, Reichenburg, Switzerland) a few hours post-mortem. The ears were kept at low temperature in the slaughterhouse and then transported in a cold box before being stored in a refrigerator at 4 °C prior to sample preparation.

The same preparation procedure was applied to all samples to gain higher reproducibility. First, the fresh ears were gently cleaned with deionized water and immediately dried with soft tissue. As the middle area at the back of the auricular presents the most stable thickness ratios of the skin layers^41^, circular piece with a diameter of 3.5 cm were punched from a fixed middle zone. The visible hairs were trimmed as close to the skin as possible using a small scissors. Then the skin tissue was carefully separated from the cartilage with a scalpel. Only skin from the back side of the ear was used throughout all experiments.

**Figure 3:**
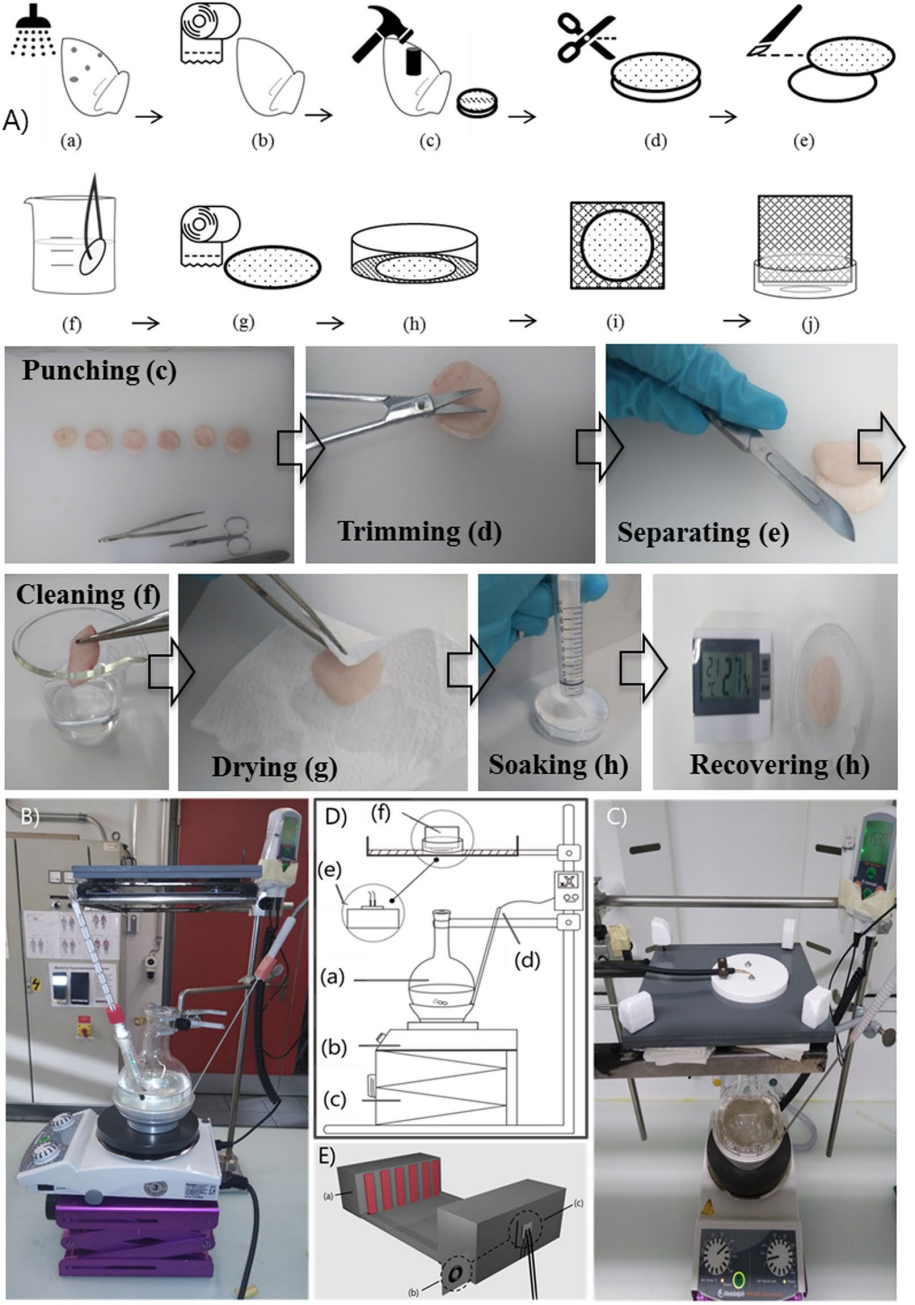
(**A**) Illustrated procedures for sample preparation and measurements. (a) Washing of the ear (b) Drying of the ear (c) Punching sample (d) Trimming the hair (e) Separating skin from cartilage (f) Cleaning sample (g) Drying sample (h) Leave the sample to recover (i) Attaching sample to plastic foam (j) Fitting into skin sample holder. Illustration of the test apparatus for calibration (**B**) and skin sample steam exposure (**C**) with description of the test apparatus (**D**): (a) Beaker (b) Hot plate (c) Lifter (d) Calorimeter system (e) Heat flux sensor (f) Sample and holder (**E**) Schematic of test apparatus for calibration and dry heat exposure: (a) Radiant heater (b) Skin sample and holder (c) Sensor.

In order to maintain a stable water hydration in the skin tissue and recover it after the sample preparation procedure, skin samples were washed again with deionized water and dried with soft tissue. For recovery, a filter paper soaked with phosphate buffered saline solution was placed in an uncovered petri dish. The skin samples, with the fat side downward, were placed onto the filter paper. Then the skin samples stayed under controlled laboratory condition [temperature of (22.0 ± 0.8 °C) and relative humidity of (27.3 ± 0.5%)] for a fixed period of recovery time. In preliminary experiments, a recovery time of 1 ~ 1.5 h was found to be appropriate, considering the exposure and observation time. 1 h of recovery time was finally chosen. After recovery, the skin sample was attached to the surface of a plastic foam cube (4 cm) to relax to a natural size. For steam exposure conditions, they were then fitted into a sample holder. For the purpose of clarity, the sample preparation procedure is schemed in Fig. 3A.

### Water and steam exposure

For water exposure, in order to compare the results with *in vivo* human skin experiments, procedures developed in our laboratory were used^6^. A water patch filled with 20 µl of water was applied to the porcine ear skin sample for controlled exposure time. Here, three exposure times, 5 min, 15 min, and 30 min, were selected.

For steam exposure, two configurations have been previously reported. One involved a chamber with a steam atmosphere^42,43^; the other was based on free steam introduced by a steam jet^44^ or tube^19^. In our study, the free steam was created by using a long neck beaker (Fig. 3B–D). This overall configuration was based on ISO 9151 with the only change being the heat source. The beaker was filled with 325 ml of water (joint opening size of 29/32, volume of 500 ml). A hot plate combined with the calorimeter system was used as a heat source. Below the hot plate, a lifter was used to adjust the distance to obtain the defined heat intensity. A PVC plate with a hole of 5 cm in diameter was designed to hold the sensor or the skin samples.

For calibration, a copper plate sensor was placed above the sensor/sample holder to define the heat intensity. The heat flux was calculated by the mass of copper disc, the specific heat capacity of the copper, the rate of rise in disc temperature and the disc area, as is done in the standard ISO 9151. In our first experiments, we tried several low levels of heat flux which are the main situations where steam burn is induced. A heat flux level of 5 kW/m^2^ was chosen, as it provided the most stable heat flux while also presented valid water profiles in the exposure time range of 15 s to 5 min.

For moisture calibration, 1.5 g of silica gel (approximately the weight of the pig skin sample) were put into a petri dish (with a hole of 2 cm diameter in the middle) and placed above the sensor/sample holder. With our defined system, the heat intensity was (4.33 ± 0.31) kW/m^2^ and the moisture absorbed by silica gel (13.31 ± 1.69) mg/min.

During steam exposure, the skin sample together with the plastic foam were placed into the petri dish (with a hole of 2 cm diameter in the middle) and then on the sensor/sample holder. Four exposure times were used, 15 s, 1 min, 3 min, and 5 min. Three repetitions were conducted for each of the exposure times. The samples were weighted before and after the exposure.

### Confocal Raman Microspectrometer

The depth profiles of water were obtained by using the 3510 Skin Composition Analyzer (SCA), an inverted confocal Raman microspectrometer designed by River Diagnostics (Rotterdam, The Netherlands). The high wavenumber region (2400–4000 cm^−1^) obtained from a laser excitation at 671 nm was used to evaluate skin hydration. For each depth profile, Raman spectra (1 s) were acquired using a step size of 2 μm from 0 μm to 60 μm, and a step size of 5 μm from 60 μm to 150 μm. Five depth profiles were recorded for each skin samples at different locations. During the spectral acquisition, skin samples were gently pressed on the acquisition window using a small stainless steel plate of 5 mm in thickness. For spectral preprocessing and water content analysis, proprietary RiverICon acquisition software and Skin Tools 2.0 software were used. Thickness of the SC was calculated from each water profile based on a Weibull curve^45^ using a Matlab algorithm (Matlab R2014b, MathWorks). Detailed information can be found in^6^.

### Dry heat exposure

Dry heat exposure was conducted to compare the water loss of samples in dry and steam conditions. Figure 3E illustrates the test apparatus for dry heat exposure. The whole setup is based on the ISO 6942 standard. A radiant heat panel is used as a heater. The distance between the radiant heater and the sensor is adjustable to get the exact same heat intensity as with the steam. After calibration, the skin sample and a filter paper with a hole of 2 cm diameter were attached on the sensor panel surface. Three repetitions were conducted for each of the exposure times. The samples were weighted before and after exposure.

### Microscope

In order to obtain a reference of the interface of epidermis and dermis, the thickness of the epidermis was determined by fluorescence microscopy (Observer A1, Zeiss). Porcine skin samples were fixed in 4% buffered formalin solution and then washed before the cross-section preparation. A skin slice was taken using the scalpel, and 3 cross-sections were made from each skin sample. 10 measurements were made for each cross-section, after which the mean thickness of epidermis was calculated.

## Electronic supplementary material

Supplementary Information
