# Supplementary material for: Prediction of Steam Burns Severity using Raman Spectroscopy on ex vivo Porcine Skin
Source: Sci Rep. 2018 May 2;8:6946. doi: 10.1038/s41598-018-24647-x (PMC5932075; doi:10.1038/s41598-018-24647-x)
Supplement: Supplementary file 1 — Supplementary Information [file 41598_2018_24647_MOESM1_ESM.docx]

**SUPPLEMENTARY INFORMATION**

**Prediction of Steam Burns Severity using Raman Spectroscopy on *ex vivo* Porcine Skin**

Lina Zhai^1,2^, Christian Adlhart^3^, Fabrizio Spano^2^, Riccardo Innocenti Malini^2^, Agnieszka K. Piątek^2^, Jun Li^1,4^ and René M. Rossi^2^

^1^Protective Clothing Research Center, College of Fashion and Design, Donghua University, 200051, Shanghai, China

^2^Empa, Swiss Federal Laboratories for Materials Science and Technology, Laboratory for Biomimetic Membranes and Textiles, CH-9014, St. Gallen, Switzerland

^3^Institute of Chemistry and Biotechnology, Zurich University of Applied Sciences, ZHAW, CH-8820, Wädenswil, Switzerland

^4^Key Laboratory of Clothing Design & Technology, Ministry of Education, Shanghai 200051, China

Correspondence should be addressed to R. M. Rossi (rene.rossi@empa.ch)

**（a） （b）**

**Figure S1 | Typical baseline corrected Raman spectra of human skin and porcine ear skin before steam exposure.** (**a**) Surface (0 μm) (**b**) Viable epidermis (40 μm)

The typical baseline corrected Raman spectra profiles of human skin and porcine ear skin before exposure are shown in Fig.S1. The CH-vibration signals for protein (2910 - 2966 cm^-1^) and OH-vibration signals (3350 - 3550 cm^-1^) for water are similar with that of human skin^1^. This further supported the observation of Tfaili et al^2^. They found that Raman vibrations at 1047 cm^-1^ and 1526 cm^-1^ were the only vibrations that presented significant differences. As the water content is calculated^3^ based on the range of 2910 - 3550 cm^-1^, the porcine skin is viewed as a good substitute for human skin.

**Figure S2 | Change in porcine skin SC thickness after different steam exposure times**

There is an approximately linear increase of SC thickness during steam exposure. SC thickness increased from (20.7 ± 2.1) μm to (24.6 ± 1.8) μm (increased by 3.9 μm) after 3 min;

**Reference**

1. Dąbrowska, A. K. *et al.* In vivo confirmation of hydration-induced changes in human-skin thickness, roughness and interaction with the environment. *Biointerphases* **11,** 31015 (2016).

2. Tfaili, S. *et al.* Confocal Raman microspectroscopy for skin characterization: a comparative study between human skin and pig skin. *Analyst* **137,** 3673–3682 (2012).

3. Egawa, M., Hirao, T. & Takahashi, M. In vivo estimation of stratum corneum thickness from water concentration profiles obtained with Raman spectroscopy. *Acta Derm. Venereol.* **87,** 4–8 (2007).
